# Comparative analysis of outcomes following craniotomy and expanded endoscopic endonasal approach resection of tuberculum sellae meningiomas: a single-institution study

**DOI:** 10.3389/fneur.2023.1139968

**Published:** 2023-05-16

**Authors:** Zhenguang Feng, Chuzhong Li, Lei Cao, Chunhui Liu, Ning Qiao, Wentao Wu, Guofo Ma, Songbai Gui

**Affiliations:** ^1^Department of Neurosurgery, Beijing Tiantan Hospital, Capital Medical University, Beijing, China; ^2^Department of Neurosurgery, Tianjin First Central Hospital, Tianjin, China; ^3^Beijing Neurosurgical Institute, Capital Medical University, Beijing, China; ^4^Department of Neurosurgery, Peking University Third Hospital, Beijing, China

**Keywords:** craniotomy, EEEA, visual outcome, extent of resection, progression-free survival, tuberculum sellae meningiomas

## Abstract

**Background:**

Traditionally, supratentorial craniotomy has been used to sever tuberculum sellae meningiomas (TSMs), but there has been a remarkably increasing tendency of extended endoscopic endonasal approach (EEEA) used to treat TSMs in the recent decade. Several documents have described the advantages and disadvantages of both approaches, but there is no consensus on whether one is superior to the other.

**Objective:**

This study aimed to compare surgical outcomes between craniotomy and EEEA for TSMS treated at our institution.

**Methods:**

From January 2015 to December 2021, a total of 84 cases of TSMs were included in this study. Cases were separated into two groups: the craniotomy group and the EEEA group. Their anamneses and surgical records were reviewed. Demographic data, presenting symptoms, tumor volume, extent of resection, visual outcomes, and follow-up data were tabulated. The Kaplan–Meier curves were constructed for the PFS for both cohorts.

**Results:**

Complete data were available for 84 surgeries; 39 cases were treated via craniotomy, and 45 were treated via EEEA. Patient demographic data, pre-operative symptoms, and tumor characteristics were similar between the two cohorts. The extent of resection was similar between the two groups (GTR: 91.11% EEEA vs. 87.18% craniotomy; STR 8.89 vs. 12.82%, *p* = 0.91). There was no difference in visual outcomes between both groups (92.1 vs. 84.84%, *p* = 0.46). An increased rate of cranial nerve injury was noted in the craniotomy group (0 vs. 10.25%, *p* = 0.04). Post-operative CSF leak rate occurred in one patient in the EEEA group. The PFS curves (*p* = 0.52) and recurrence/progression rates (13.33 vs. 20.51%, *p* = 0.39) were similar between the two groups.

**Conclusion:**

Both EEEA and craniotomy can successfully sever TSMs. The recurrence/progression rate and PFS appear to be similar between the two groups. Although there are no differences in EOR and visual outcomes between the two groups, there was a clear trend in the EEEA group to obtain a better outcome. CSF leakage was common in the EEEA cohort, whereas the rate of cranial nerve injury was found to be higher in the craniotomy cohort. We believe that our data support the conclusion that EEEA surgery is the preferred approach for the removal of TSMs.

## Introduction

Tuberculum sellae meningiomas (TSMs) are regarded to be a group of “suprasellar meningiomas” which commonly originate from the dura of the tuberculum sellae, chiasmatic sulcus, planum sphenoidale, diaphragma sellae, and anterior clinoid process (1). They approximately represent 5–10% of intracranial meningiomas (2). Due to the special anatomical position, TSMs are often surrounded by important neurovascular structures, such as the optic apparatus, pituitary stalk, internal carotid artery, and anterior communicating artery complex, which make surgical resection difficult.

Traditionally, supratentorial craniotomies including the pterional, frontolateral, unilateral subfrontal, and bilateral subfrontal approaches have been used to sever these tumors (3–7). However, these procedures required a significant amount of brain retraction and manipulation of neurovascular structures, which commonly resulted in brain contusion and neurovascular injury. In recent years, with the improvement of endoscopic instruments and progress in surgical skills, the expanded endoscopic endonasal approach (EEEA) has become increasingly popular for the treatment of TSMs (8–12), via transplanum extensions, and this approach provides direct access to the infrachiasmatic, olfactory, and subfrontal regions.

To investigate the issue, we directly compared the visual prognosis, extent of resection (EOR), complication rate, and progression-free survival (PFS) between both groups who were treated by craniotomy or EEEA at a single center.

## Methods

All patients with midline suprasellar lesions that were resected at our hospital between January 2015 and December 2021 were identified by a retrospective review of the medical chart. Among them, patients with histologically verified pathologies of meningiomas were included in the study. The enrolled patients were arranged into two groups according to the surgical approach: the craniotomy group and the EEEA group. For each case, anamnesis, surgical log, and surgical video were reviewed. Their demographics, signs, and symptoms at diagnosis, tumor volumes, visual outcomes, EOR, and the relationship of the tumor to the optic canal and PFS were obtained. Radiographic data were collected from the picture archiving and communication system (PACS). Tumor volumes were computed using the following equation: V = (A × B × C)/2, where A, B, and C represent the dimensions of the tumor in three orthogonal planes. The study was performed under an institutional review board-approved protocol in compliance with the regulations set by our institution for the study of human subjects with their informed consent.

### Radiological assessment

All patients underwent a complete pre-operative radiological examination including MRI and CT scan. An enhanced MRI was performed in order to assess the tumor size, shape, optic canal invasion, lateral extension, and study the tumor's relationship with surrounding anatomical structures, such as the internal carotid artery, pituitary stalk position, and hypothalamus. Three-dimensional reconstruction CT might supplement useful information about bone destruction at the site of origin of the tumor, anatomy of the sphenoidal sinuses, and also intratumoral calcification. Post-operative MRI was performed routinely at 3 and 12 months, and yearly thereafter. The EOR classification was as follows: (1) Gross total resection (GTR) was defined as a total resection of the tumor with no residual lesion observed in post-operative MR images, (2) subtotal resection (STR) was defined as ≥90% of the tumor was resected in post-operative MR images, and (3) partial resection (PR) was defined as <90% of the tumor was removed in post-operative MR images.

### Endocrinological assessment

A complete endocrinological assessment was undertaken pre-operatively and post-operatively for all patients, including prolactin, glucocorticoid, growth hormone, thyroid hormone, and gonadal hormone. Monitoring for diabetes insipidus was carried out by measuring urine-specific gravity, serum sodium, and fluid balance during the post-operative period.

### Ophthalmological test

All patients had formal ophthalmological examinations pre-operatively, including visual acuity and visual field. The first post-operative assessment was generally performed at discharge. During follow-up, these examinations were performed at 3 and 6 months after surgery.

### Statistics analysis

A descriptive analysis was performed for the continuous and categorical variables. Continuous variables were described using mean, median, and interquartile range (IQRs), whereas categorical variables were reported in terms of frequencies. A chi-square test or Fisher's exact test was performed to analyze the distributions of categorical variables between groups. The Kaplan–Meier curves were constructed for the PFS for both cohorts, and the curves were compared using the log-rank test. A *P*-value of < 0.05 was considered to be statistically significant. Analyses were performed with the statistical software STATA 14.0 (StataCorp., College Station, Texas).

## Results

### Patient population and clinical presentation

A total of 84 patients were included in the present study. Thirty-nine (46.43%) surgeries were performed via a craniotomy, whereas 45 (53.57%) were performed via the EEEA approach. Of the 39 patients, 22 received a pterional approach, 10 received a frontolateral approach, and seven received a unilateral subfrontal approach. Among 39 craniotomy surgeries, three (7.69%) had previously undergone surgery via a transcranial approach. Of the 45 EEEA cases, five (11.11%) had previously undergone surgery via a craniotomy or EEEA. The proportion of patients with previous surgery was not different between the two groups (7.69 vs. 11.11%, *P* = 0.91). The mean age for all patients was 53.1 years, with an IQR of 36–57 years. The average age among craniotomy (52.2 years) and EEEA (53.4 years) patients was comparable (*p* = 0.92). There was no difference in the sex ratio between the two groups (*P* = 0.9). A description of pre-operative symptoms showed that visual impairment (84.52%) was the most common, followed by headache (40.47%). The rates of pre-operative symptoms were comparable between cases treated with a craniotomy and EEEA (84.61 vs. 84.44%, *p* = 0.99; 41.67 vs. 44.19%, *p* = 0.82) (Table 1).

**Table 1:** Characteristics and symptoms of patients treated with craniotomy and EEEA.

**Variable**	**All cases**	**Craniotomy**	**EEEA**	***p*-value**
No. of cases	84	39	45	
**Age in yrs**				
Mean (SD)	53.1 (19.7)	52.2 (18.4)	53.4 (19.1)	0.92
Median (IQR)	47 (36–57)	45 (35–57)	48 (36–58)	
Female/male	11/73	5/34	6/39	0.9
**Pre-op symptoms**				
Visual impairment	71 (84.52%)	33 (84.61%)	38 (84.44%)	0.99
Headaches	34 (40.47%)	15 (38.46%)	19 (42.22%)	0.82
Previous operation	8 (9.52%)	3 (7.69%)	5 (11.11%)	0.91

### Neuroradiological findings and EOR

The mean tumor volume for the entire cohort treated with either a craniotomy or EEEA procedure was 10.97 cm^3^ with an IQR of 7.56–17.4 cm^3^ (Table 2). The average tumor volume among craniotomy (10.64 cm^3^) and EEEA (11.32 cm^3^) patients was comparable (*p* = 0.81). Fewer patients with intratumoral calcification were treated with a craniotomy compared with EEEA (43.59 vs. 46.67%, p = 0.41). The rates of the optic canal invaded by tumor were comparable between cases treated with a craniotomy and EEEA (53.84 vs. 60%, *p* = 0.59). In the craniotomy group, 34 (87.18%) cases harvested GTR and five (12.82%) achieved STR. In the EEEA group, 41 (91.11%) harvested GTR and four (8.89%) achieved STR. No significant difference was found in the EOR between the two cohorts (*p* = 0.56) (Table 2).

**Table 2:** Characteristics in tumors resected and surgical outcomes via craniotomy and EEEA.

**Variable**	**All cases**	**Craniotomy**	**EEEA**	***p*-value**
No. of cases	84	39	45	
Pre-op tumor vol in cm^3^ (SD)	10.97 cm^3^	10.64 cm^3^	11.32 cm^3^	0.81
Intratumoral calcification	38 (45.24%)	17 (43.59%)	21 (46.67%)	0.41
optic canal invaded by tumor	48 (57.14%)	21 (53.84%)	27 (60%)	0.59
**EOR**				
GTR	75 (89.28%)	34 (87.18%)	41 (91.11%)	0.56
STR	9 (10.72%)	5 (12.82%)	4 (8.89%)	
**Visual outcome** ^&^				
Improvement		28 (84.84%)	35 (92.1%)	0.46
Stable		5 (15.16%)	3 (7.89%)	

& Patients with pre-operative visual impairment.

### Visual outcome

In the EEEA group, no patients experienced deterioration of vision post-operatively. Among 38 patients with visual impairment prior to surgery, we found improvement of the visual defect in 35 (92.1%) patients post-operatively and at follow-up. The visual was stable in the other three patients. In the craniotomy group, two patients experienced transient deterioration of vision post-operatively. Among 33 patients with visual impairment prior to surgery, the improvement in vision occurred in 28 (84.84%) cases. No significant difference was found in the visual outcome between the two cohorts (92.1 vs. 84.84%, *p* = 0.46) (Table 2).

### Complications and follow-up

Pre-operative endocrinological assessments were normal in all patients. In the EEEA group, five patients had transient hypocortisolism and two patients presented transient diabetes insipidus post-operatively. In the craniotomy group, three patients had transient hypocortisolism post-operatively. In the EEEA group, cerebrospinal fluid (CSF) leakage occurred in one patient who experienced lumbar drain and absolute bed rest, but repair operations were performed 1 week later. The incidence of cranial nerve palsy (oculomotor nerve) was significantly higher in the craniotomy group (10.25 vs. 0%, *p* = 0.04).

**Figure 1:**
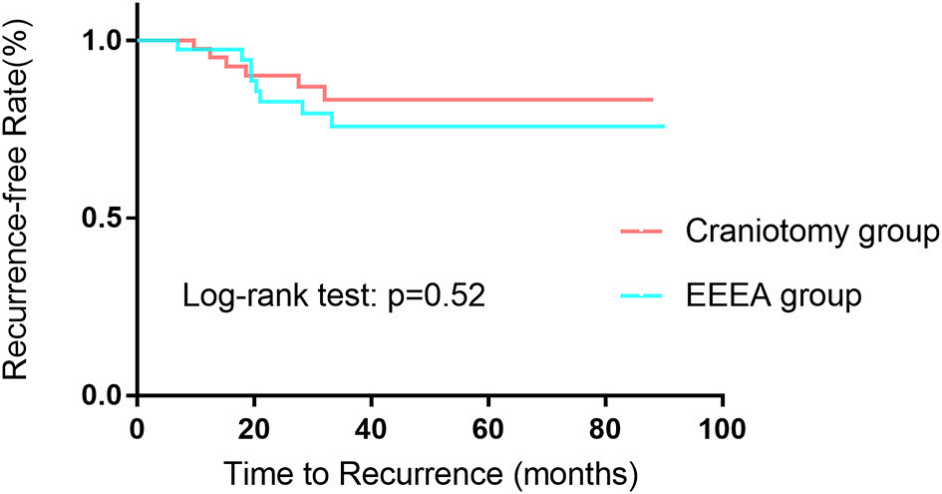
Kaplan–Meier curves comparing PFS for patients undergoing craniotomy vs. EEEA for TSMs.

The mean follow-up time in the craniotomy group was 1,494 days, and the mean follow-up time in the EEEA group was 1,296 days (*p* = 0.2). The tumor recurrence or progression rate was comparable between the craniotomy group and the EEEA group (20.51 vs. 13.33%, *p* = 0.39) (Table 3). The mean PFS times were similar between the two cohorts (1,244 vs. 1,154 days, *p* = 0.56). In the 14 cases with tumor recurrence or progression, 11 (78.57%) underwent radiotherapy and three (21.43%) underwent a reoperation. In addition, Kaplan–Meier curves were constructed for the PFS for both cohorts (Figure 1). There was no significant difference in PFS curves for the craniotomy and EEEA cohorts based on the log-rank test (*p* = 0.52).

**Table 3:** Complications, follow-up time, PFS, and recurrence/progression rates for patients treated with craniotomy and EEEA.

**Variable**	**All cases**	**Craniotomy**	**EEEA**	***p*-value**
No. of cases	84	39	45	
**Post-operative complications**				
Transient hypocortisolism	8 (9.52%)	3 (7.69%)	5 (11.11%)	0.72
Transient diabetes insipidus	2 (2.38%)	0	2 (4.44%)	0.49
CSF leakage	1 (1.19%)	0	1 (2.22%)	>0.99
Cranial nerve palsy	4 (4.76%)	4 (10.25%)	0	
**Follow-up**				
Mean follow-up time in days		1,494	1,296	0.2
Mean follow-up time in days		1,244	1,154	0.56
Tumor recurrence or progression	14 (16.67%)	8 (20.51%)	6 (13.33%)	0.39

## Discussion

Tuberculum sellae meningiomas (TSMs) are a common kind of benign lesions in the sellae region. Their specific anatomical location frequently causes the impairment of visual acuity and field and the difficulty in surgical resection. Hence, improvement of visual function and achieving GTR of the tumor are the main goals of the treatment of TSMs.

Historically, supratentorial craniotomies as mature approaches have been the main method for the treatment of TSMs. However, these surgical approaches require neurosurgeons to create surgical corridors to access the suprasellar region, which commonly results in high perioperative complications. In the last decade, there has been an increasing tendency for EEEA used to treat TSMs. It is generally assumed that EEEA has several advantages over traditional transcranial approaches, including (i) a direct visualization of the undersurface of the optic nerves and chiasm; (ii) offering a panoramic surgical view to minimizing the possibility of residual tumor; and (iii) direct access to the tumor avoiding brain retraction and repeated manipulation between neurovascular structures. Although there are no clear criteria to help the surgeon decide between the transcranial and endoscopic approaches, it is generally assumed that tumors with extensive dural attachment, vascular encasement, and lateral extension are more suitable for open approaches (13–15), but now, the features also gradually become relative contraindications of EEEA, and many neurosurgeons with rich endoscopic experience try to use EEEA to remove tumors with these features. In the study, we compared the surgical outcomes between the two groups who were treated by craniotomy or EEEA in our center.

### Visual outcome

Visual impairment was the most common presentation in patients with TSMs. In our cohort, 71 (84.52%) patients presented with blurred vision and visual field. The ratio was similar to previous reports (5, 6, 16, 17). The majority of patients could achieve improvement in visual function after surgery. In our study, the visual acuity improvement rates were 84.61% in the craniotomy group and 84.44% in the EEEA group, respectively. The data were also covered by the published literature. Several reports compared the visual outcome between the endoscopic group and the transcranial group, and the results showed that the endoscopic group could obtain a higher improvement rate of visual function after surgery. Sankhla et al. (16) reported that in their case series, a significantly higher rate of visual improvement was observed in the endoscopic group as compared to the open group (96 vs. 79%); meanwhile, visual worsening after surgery was greater in the open group (21 vs. 4%). Divitiis et al. (18) reported that 71.4% of patients had improved vision and 28.6% of patients had no deterioration of vision in the endoscopic group, but in the transcranial group, 61.4% of patients had improved vision, 25% of patients had an unchanged vision, and 13.6% of patients had deterioration of vision. Bander et al. (17) reported that 93% of EEEA patients experienced improved or stable visual outcomes, but 56% of patients were in the open group. The difference had a statistical significance between the two groups (*p* = 0.049). A recent meta-analysis also showed better visual outcomes with EEA (77.7 vs. 60.7%, *p* < 0.01) although it had some limitations, including inter-group approach selection biases (19). In our study, although no significant differences were found in the visual outcomes between the EEEA and craniotomy groups, there was a clear trend in the EEEA group to obtain a better visual outcome. We considered that EEEA had several natural advantages to improving visual function compared to the open approach in the treatment of TSMs. First, for the patients with the tumor-invaded optic canal, EEEA had the possibility of a 270° early decompression of the optic canal, which was not only good for the improvement of visual impairment but also reduced the tumor recurrence. In the study, tumor invasion of the optic canal occurred in 11 patients. Of these patients, the optic canal was fully opened intraoperatively and the intracanalicular tumor was removed. Second, approaching the tumor from below allowed better visualization and preservation of the superior hypophyseal and anterior cerebral artery which supplied the chiasma from below and above. Third, under a close-up, enlarged, and high-definition view, bimanual microdissection techniques can be performed to carefully dissect the tumor away from the optic chiasm and vascular perforators with direct visualization of the surgical plane between the tumor and critical structures, which can minimize deterioration of visual caused by blind manipulation.

### Extent of resection

Although a more excellent visual prognosis could be harvested in the endoscopic group, the extent of tumor resection seemed to be similar between the two groups based on the previous reports (5, 6, 11, 16, 20). Bander et al. (17) reported that the average extent of resection achieved was not significantly different between the two groups (98.80% ± 3.32% vs. 95.13% ± 11.69%, *p* = 0.206). Kong et al. (21) reported that GTR rates and relapse-free survival were not different between the two groups although the locations of residual or recurred tumors definitely differed. In 2013, Clark et al. (9) undertook a meta-analysis. The results showed that there were no differences in the rate of gross total resection or perioperative complications between the two groups. In another meta-analysis by Jimenez et al. (20), they also obtained the same result. In our case series, the GTR was 91.11 and 87.18% in the EEEA group and the craniotomy group, which was similar to previous literature. There was no statistical significance in EOR between both groups. Nonetheless, we considered there were still advantages of EEEA for the treatment of TSMs except for the abovementioned ones, including (i) accessing the tumor from below facilitates dealing with the tumor base to devascularize the tumor early and (ii) eroded skull base bone and dural by tumor are easier to be processed through EEEA to reduce the possibility of tumor recurrence.

### Surgical complications

In the past, cerebrospinal fluid (CSF) leakage was a common and serious complication after neuroendoscopic surgery, which could result in secondary tension pneumocephalus and meningitis and significantly limited the development of neuroendoscopic skill. Since a vascularized septal mucosal flap was applied to the skull base reconstruction, the prevalence of CSF leakage after endoscopic transnasal surgery has been reduced considerably (20, 22–24). Yu et al. (11) reported that the incidence of CSF leakage was 7.5% in their case series after EEEA. Mccoul et al. (25) and Hadad et al. (26) reported the incidences of CSF leakage were 3.1 and 5%, respectively. In our patients, one patient (2.22%) experienced CSF leakage post-operatively. The ratio was similar to the report by Mccoul et al. (25).

Mou et al. (27) proposed that vascularized pedicled septal flaps and *in situ* bony flaps in skull base reconstruction were more conducive to reducing the occurrence of cerebrospinal fluid leakage. In our cohort, the transient hypocortisolism transient diabetes insipidus occurred in 10 patients. We considered that the phenomenon was related to the disturbance of the pituitary stalk. In addition, two patients experienced transient deterioration of vision post-operatively in the craniotomy group, which might result from the traction of the optic nerve.

## Limitations

A potential confounding factor in a retrospective study of this nature is selection bias. For example, tumors with a more lateral extension might tend to be selected for craniotomy, whereas more midline tumors would be selected for EEEA. In turn, this could affect results, such as EOR. In the future, a case-matched study is worth designing and executing.

## Conclusion

Both EEEA and craniotomy can successfully sever TSMs. The recurrence or progression rate and PFS appear to be similar between the two groups. Although there are no differences in EOR and visual outcomes between the two groups, there was a clear trend in the EEEA group to obtain a better outcome. CSF leakage was common in the EEEA cohort, whereas the rate of cranial nerve injury was found to be significantly higher in the craniotomy cohort. We believe that our data support the conclusion that EEEA surgery is the preferred approach for the removal of TSMs.

## Data availability statement

The raw data supporting the conclusions of this article will be made available by the authors, without undue reservation.

## Ethics statement

The studies involving human participants were reviewed and approved by the Ethics Committee of Beijing Tiantan Hospital. The patients/participants provided their written informed consent to participate in this study.

## Author contributions

Conception and design: SG and ZF. Data collection: WW and NQ. Analysis and interpretation of data: ZF and GM. Drafting the article: ZF, SG, and CLiu. Approved the final version of the manuscript on behalf of all authors: SG. Critically revising the article and study supervision: all authors.
